# Structural racism and health: Assessing the mediating role of community mental distress and health care access in the association between mass incarceration and adverse birth outcomes

**DOI:** 10.1016/j.ssmph.2023.101529

**Published:** 2023-10-04

**Authors:** Anders Larrabee Sonderlund, Natasha J. Williams, Mia Charifson, Robin Ortiz, Shawnita Sealy-Jefferson, Elaine De Leon, Antoinette Schoenthaler

**Affiliations:** a Center for Healthful Behavior Change, Institute for Excellence in Health Equity, NYU Grossman School of Medicine, USA; b Research Unit of General Practice, Department of Public Health, University of Southern Denmark, Denmark; c Department of Population Health, NYU Grossman School of Medicine, USA; d Vilcek Institute of Graduate Biomedical Sciences, NYU Grossman School of Medicine, USA; e Department of Pediatrics, NYU Grossman School of Medicine, USA; f College of Public Health, Ohio State University, USA

**Keywords:** Incarceration, Birth outcomes, Pregnant, Structural racism, Reproductive health

## Abstract

Research has linked spatial concentrations of incarceration with racial disparities in adverse birth outcomes. However, little is known about the specific mechanisms of this association. This represents an important knowledge gap in terms of intervention. We theorize two pathways that may account for the association between county-level prison rates and adverse birth outcomes: (1) community-level mental distress and (2) reduced health care access. Examining these mechanisms, we conducted a cross-sectional study of county-level prison rates, community-level mental distress, health insurance, availability of primary care physicians (PCP) and mental health providers (MHP), and adverse birth outcomes (preterm birth, low birth weight, infant mortality). Our data set included 475 counties and represented 2,677,840 live U.S. births in 2016. Main analyses involved between 170 and 326 counties. All data came from publicly available sources, including the U.S. Census and the Centers for Disease Control and Prevention. Descriptive and regression results confirmed the link between prison rates and adverse birth outcomes and highlighted Black-White inequities in this association. Further, bootstrap mediation analyses indicated that the impact of spatially concentrated prison rates on preterm birth was mediated by PCP, MHP, community-level mental distress, and health insurance in both crude and adjusted models. Community-level mental distress and health insurance (but not PCP or MHP) similarly mediated low birthweight in both models. Mediators were less stable in the effect on infant mortality with only MHP mediating consistently across models. We conclude that mass incarceration, health care access, and community mental distress represent actionable and urgent targets for structural-, community-, and individual-level interventions targeting population inequities in birth outcomes.

## Highlights

• Racialized mass incarceration contributes to racial inequities in adverse birth outcomes.
• The mechanisms of this association have never been empirically assessed.
• We find that the association is mediated by community-level mental stress and health care access.
• This has policy implications in terms of uprooting structural racism and achieving health equity.

## 1 Introduction

Population rates of adverse birth outcomes are about twice as high in the United States (U.S.) as in most other developed countries (Thakrar et al., 2018). In 2020, preterm birth (PTB) (<37 weeks gestational age) and low birthweight (LBW) (<2500 g at birth) affected 10.5% and 8.2%, respectively, of all US births (CDC, 2021; Valenzuela, 2022). Moreover, the incidence of adverse birth outcomes disproportionately affects minoritized populations. Specifically, in 2020, PTB and LBW rates for non-Hispanic Black (hereafter ‘Black’) pregnant women were 14.4% and 14.2%, respectively (Thakrar et al., 2018). These well-documented disparities have persisted through recorded history and recently begun to widen further at the national level (Thakrar et al., 2018).

Mounting evidence shows that these disparities in reproductive health outcomes are multifactorial and largely due to structural racism (Bishop-Royse et al., 2021; Krieger et al., 2020; Larrabee Sonderlund et al., 2022a). Structural racism may be defined as the multi-faceted macro-level societal forces that are rooted in the ideology of White supremacy and which produce and maintain racial inequities in opportunity, resources, power, and well-being (Bailey et al., 2017, 2021; Delgado et al., 2023; Gee et al., 2011). To date, the literature has predominantly focused on de facto racialized economic and residential segregation as a key manifestation of structural racism that impacts negatively and disproportionately on birth outcomes among people from minoritized populations (Chambers et al., 2018; Janevic et al., 2020, 2021a, 2021b; Massey et al., 2014; Odoms-Young, 2018). This association appears to occur primarily through socio-economic and environmental pathways (e.g., by restricting access to pre- and post-natal care, increased environmental exposures) (Bishop-Royse et al., 2021; Chambers et al., 2019; Janevic et al., 2021b; Larrabee Sonderlund et al., 2022b; Massey et al., 2014). Recent evidence, however, suggests that the ongoing and disproportionate mass incarceration of Black people represents an additional and closely-linked indication of structural racism that also contributes to disparities in population-level adverse birth outcomes (Dyer et al., 2019; Larrabee Sonderlund et al., 2022a; Pabayo et al., 2019; Sealy-Jefferson et al., 2020; Yi et al., 2021).

As of 2019, 2.1 million people were either in jail or prison in the U.S. – a prevalence of 810 per 100,000 adults. This rate is higher than anywhere else in the developed world (Brief, 2020; Minton, G, & Zeng, 2019). The disparities in U.S. incarcerations are also deeply startling, with Black people comprising over 40% of the incarcerated population, but only 13% of the total U.S. population. By contrast, White people represent 57% of the general population, but only 39% of incarcerated persons (Sawyer, 2020a, 2020b). Research indicates that these inequities are driven primarily by deep-rooted biases in the criminal justice system that discriminate against people from minoritized groups and manifest as punitive policing strategies and laws, prejudiced systems of bail and plea bargaining, as well as inequities in conviction rates and sentencing (Alexander, 2010; Commission, 2018).

Incarceration has extensive health implications not only for the individual, but also for their immediate family (children, spouses, parents) – including increased risk of adverse birth outcomes (Alexander, 2010; Turney, Schnittker, & Wildeman, 2012; Yi et al., 2021). This association appears to be due to the financial instability, decreases in emotional and practical support, social isolation, and amplified stress that often result from having a family member removed to jail or prison (Clear, 2009; Drucker, 2013). Importantly, however, recent studies have found that when incarceration rates are spatially concentrated (e.g., in a neighborhood or county), it may be negatively associated with the health of the surrounding community at large (Dumont et al., 2012; Escobar et al., 2020; Hatzenbuehler et al., 2015; Kajeepeta et al., 2020, 2021; Topel et al., 2018). In terms of adverse birth outcomes specifically, Sealy-Jefferson et al. (Sealy-Jefferson et al., 2020) and Holaday et al. (Holaday et al., 2023) found associations between residence in neighborhoods impacted by mass incarceration and increased risk of adverse birth outcomes – particularly among Black pregnant people. Dyer et al. (Dyer et al., 2019) demonstrated a similar pattern in Louisiana where parish-level jail-incarceration rates were positively associated with PTB among Black residents. Comparable associations have been reported at the state level as well (Conway, 2021). Taken together, these studies indicate that mere residence in an area affected by mass incarceration may put the individual at increased risk of experiencing adverse birth outcomes. Given that incarceration rates cluster in low-income majority-Black areas, it follows that the associated population health risks will be borne disproportionately by this demographic as well.

### 1.1 Rationale and hypotheses

While existing research supports an overall positive association between spatial concentrations of incarceration and adverse birth outcomes, none explicitly examine the pathways that may account for this association. This knowledge, however, is crucial for targeting interventions and uprooting structural barriers to population health equity. Bridging this gap in the evidence base, we investigate the underlying mechanisms of the relation between spatially concentrated mass incarceration and adverse birth outcomes. Further, in examining the community-level impacts, we are compelled to reframe the discussion from an individual focus on corrective behavior and/or recidivism to structural and political investments, including potential federal and state policy.

**Fig. 1:**
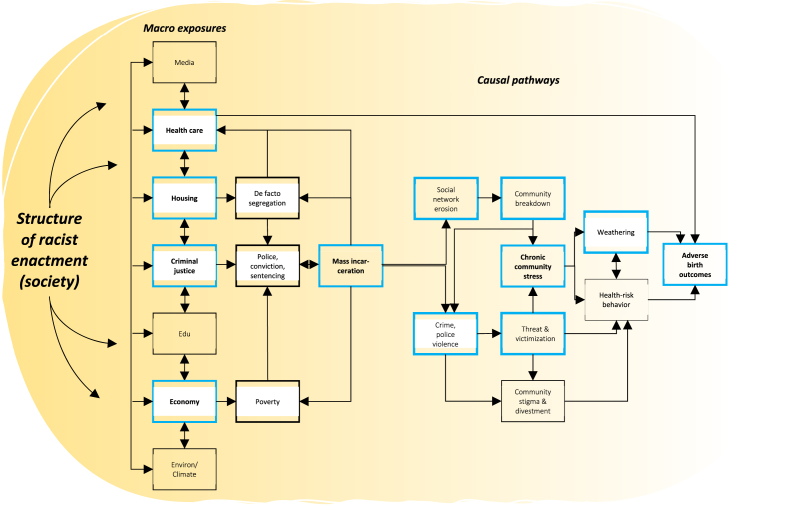
Pathway model (blue boxes) of the impact of racialized mass incarceration on adverse birth outcomes (Larrabee Sonderlund et al., 2022a). (For interpretation of the references to colour in this figure legend, the reader is referred to the Web version of this article.)

Previously, we proposed two theoretical pathways through which spatial concentrations of prison rates may impact on adverse birth outcomes (Larrabee Sonderlund et al., 2022a). One relates to the proliferation of psychosocial stress – a known risk factor for reproductive health – that communities affected by mass incarceration experience (Fig. 1) (Larrabee Sonderlund et al., 2022a). Here, the social capital and support structures that underpin healthy, cohesive, and resilient communities may fragment and deteriorate under the weight of localized mass imprisonment of community members. In turn, this increases population vulnerability to psychophysiological stress effects (e.g., weathering, allostatic load) and associated health consequences, including pre- and post-natal adverse health outcomes (Hickson et al., 2022; Topel et al., 2018). The other theorized pathway pertains to the lack of healthcare access. When a residential area is stigmatized as a ‘high-incarceration area’, this may have repercussions for the availability of a range of critical resources, including quality primary health care and insurance – the lack of which represents a clear risk factor for adverse birth outcomes (Jahn et al., 2022; Janevic et al., 2020, 2021b; Larrabee Sonderlund et al., 2022a). Further, in most states when community members are removed from their homes to prison, they are acknowledged in the national census as residents in the area in which they are imprisoned (i.e., *prison gerrymandering*) and not in the area of their actual residential address and community membership. Spatial concentrations of incarceration thus significantly skew census demographic counts, eroding political representation and clout in affected areas. In turn, this may divert the flow of federal and state resources (e.g., health care) away from communities affected by mass incarceration and towards areas with jails and/or prisons (Golembeski et al., 2008).

Based on this work, we now propose to examine these theoretical pathways. Specifically, we use secondary county-level prison and health data from multiple sources to test the following hypotheses (H_X_):

H1 County-level prison rates are positively associated with county-level rates of LBW, PTB, and infant mortality (IM).

H2 The association between county-level prison rates and LBW, PTB, and IM is mediated by lack of health care access.

H3 The association between county-level prison rates and LBW, PTB, and IM is mediated by increased community-level mental stress.

Finally, we note that given the large body of evidence on the deeply-rooted Black-White inequities in incarceration and adverse birth outcomes, we do not include hypotheses explicitly related to race differences. Rather, we focus on the manifestation of structural racism (in this paper, mass incarceration) and propose to understand the mechanisms by which it may explain increased rates of adverse birth outcomes in Black populations.

## 2 Methodology

### 2.1 Research design

We conducted a lagged cross-sectional study using county as the unit of analysis. Data from 2014 to 2016 were sourced from multiple data sets and linked by Federal Information Processing Standards (FIPS) county codes.

### 2.2 Measures & data sources

#### 2.2.1 Primary outcome – county-level rates of adverse birth outcomes

Our primary outcome was county-level rates of adverse birth outcomes, operationalized as three separate variables – LBW, PTB, and IM. Consistent with recommendations in the International Statistical Classification of Diseases, 11th Revision (ICD-11) (WHO, 2023), LBW was defined as full-term live births weighing 2,500 g or less. PTB included live births occurring at earlier than 37 weeks of gestation, calculated based on obstetric estimate of gestation at delivery (National Center for Health Statistics (NCHS) guidelines) (WHO, 2023). IM was defined as death of an infant before their first birthday (WHO, 2023). We obtained annual county-level frequency data for all three outcomes from the NCHS Vital Statistics database for 2016 (CDC, 2014-2016a). These data included only singleton births. Due to privacy constraints, PTB, LBW, and IM counts of less than 10 for a given county were suppressed. Finally, to reduce estimation biases for counties with small populations, we restricted our analyses to counties that recorded at least 50 births (Chambers et al., 2018).

#### 2.2.2 Primary exposure – county-level prison rates

Our primary exposure variable was county-level prison rates per 100,000 population. Nationwide county-level prison rates for 2014 were obtained from the Vera Institute of Justice (VIJ) public data libraries (VIJ, 2023). These data were originally sourced from the National Corrections Reporting Program and represent the number of individuals sentenced to the state prison authority (VIJ, 2020). The data was aggregated by county of commitment (i.e., the county in which the individual was charged with a crime), which typically corresponds to the individual's county of residence (VIJ, 2023), and further stratified by two gender categories (man/woman) as well as by racial and ethnic groups (we retained data for Black and White populations). Annual county-level prison rates per 100,000 county residents aged 15–64 were then calculated based on the number of individuals incarcerated in prison on December 31, 2014. Pertinent county population demographics (age, race, and ethnicity) were obtained by the VIJ from the NCHS (VIJ, 2020). Age limits of 15–64 were used by VIJ because people outside of this age range are at very low risk of prison incarceration. Focusing on this segment of the population thus provides a more accurate estimate of imprisonment prevalence (VIJ, 2020).

#### 2.2.3 Pathway variables

We included two categories of mediator variables in our analyses: Community-level mental distress and access to health care. For the former pathway, we used the Behavioral Risk Factor Surveillance System age-adjusted measure of *poor mental health days* for 2015 (CDC, 2014-2016b). This measure reflects the average number of days that adult residents in a given county answered to the question, “Now, thinking about your mental health, which includes distress, depression, and problems with emotions, for how many days during the past 30 days was your mental health not good?” (CDC, 2014-2016b). We included this variable based on evidence that has linked community-level mental distress with increased incarceration rates (Hickson et al., 2022) and pre- and post-natal adverse health outcomes (Farr et al., 2013; Willet et al., 2012).

Our second mediator category comprised three proxy measures of primary care availability and access. These included the percentage of the county population under 65 years of age that was uninsured (%Uninsured), as well as the ratio of county population to the number of county primary care physicians (PCP rate) and mental health providers (MHP rate). PCP data originated from the American Medical Association Physician Masterfile which contains up-to-date information on nearly all Doctors of Medicine and Doctors of Osteopathic Medicine in the nation (AHRF and Area Health Resources Files (AHRF), 2014–2016). MHP data was from the National Provider Identification (NPI) Registry (CMS, 2022), and insurance data was from the US Census Bureau. Data for all three variables reflected 2015 counts. We included these variables because increased rates of adverse birth outcomes have been associated with decreased PCP rates (Chang et al., 2011; Macinko, Starfield, & Shi, 2007; Shi et al., 2001, 2004), MHP rates (Feinberg et al., 2016; Howard et al., 2020; Witt et al., 2012), and health insurance coverage (Taylor, Liu, & Howell, 2020). Similarly, increased population incarceration rates have been linked with poorer insurance coverage and health care availability (Testa, Santos, & Weiss, 2020; Weidner et al., 2019; Zhao et al., 2023). While adding pre- and post-natal care access as a mediator would have been ideal, county-level data for this variable was unavailable.

#### 2.2.4 Control variables

Given the multidimensional and intersecting pathways that may link prison rates and population health outcomes, there are recommendations from prior literature to avoid over-controlling (Becker et al., 2016; Zuberi et al., 2008). Therefore, we only considered those factors that we deemed – based on existing evidence – plausible sources of spuriousness. These included county-level poverty (Campbell et al., 2016; Clear, 2009; Cubbin et al., 2020; Drucker, 2013), educational level (Amjad et al., 2019; Heitzeg, 2009; Hemez, Brent, & Mowen, 2020), social capital (Amjad et al., 2019; Clear, 2009; Ehsan et al., 2019), county population racial makeup (percentage Black and White), and Black/White segregation (Index of Dissimilarity). All control variables are from 2014 and selected *a priori*.

Poverty: Poverty data was sourced from Small Area Income and Poverty Estimates. We acknowledge that because economic factors represent a risk factor for, and an outcome of, incarceration (Alexander, 2010; Drucker, 2013), this variable may both moderate and/or mediate an effect on birth outcomes. For the purposes of this study, however, we included poverty as a covariate in line with previous research (Conway, 2021; Dyer et al., 2019; Jahn et al., 2020).

Education: We used high school graduation rates from EDfacts. We entered this covariate because past research indicates a (negative) association with incarceration (e.g., the *school to prison pipeline*) and adverse birth outcomes (Amjad et al., 2019; Mallett, 2016).

Social capital: The Social Capital Index (Rupasingha, Goetz, & Freshwater, 2006). This index is a composite measure of county-level civic engagement (e.g., voter turnout, political organizations, census response rate), number of faith-based and social/recreational associations (e.g., sports clubs, religious organizations), business associations and establishments (e.g., labor organizations, professional associations). The assessment data was downloaded directly from the index creators’ institutional website (Penn State College of Agricultural Sciences).

Residential segregation: Index of Dissimilarity and county population racial makeup. Index of Dissimilarity scores and county percentages for Black and White population originated from the 2014 US Census.

### 2.3 Analytic approach

Implementing a lagged design, we used prison rates from 2014 to predict mediators in 2015 and birth outcomes in 2016. To test our hypotheses, we implemented a three-step analytic approach. First, we generated descriptive statistics and correlations for our key variables. Next, we conducted unadjusted and adjusted least squares regression analyses to test H_1_ with separate analyses for each outcome (county-level PTB, LBW, and IM). Finally, mediation analyses were carried out. Mediations were conducted using the PROCESS macro (model 4) with bootstraps set at 5000 samples, generating 95% confidence intervals by sorting the lowest to the highest of bootstrap samples. The bootstrap method is based on regression analysis with direct and indirect effects derived from two linear models. One estimates the mediator *M* from the exposure *X*

*M* = *i*_*M*_ + *a*_*1*_*X* + *e*_*M*_

The other estimates the outcome *Y* from both *X* and *M*

*Y* = *i*_*Y*_ + *c’X* + *b*_*1*_*M* + *e*_*Y*_

Where *i*_*M*_ and *i*_*Y*_ are regression constants, *e*_*M*_ and *e*_*Y*_ are errors in the estimations of *M* and *Y*, and *a*, *b*, and *c’* are regression coefficients for *X* predicting *M*, *M* predicting *Y*, and *X* predicting *Y*, respectively. The indirect effect of *X* on *Y* through mediator *M* is estimated as the product of the effect of *X* on *M* and the effect of *M* on *Y* (*a*_*1*_ *b*_*1*_) (Hayes, 2012, 2022). All analyses were conducted using SPSS 25.0 and reported according to the AGReMA statement (Lee et al., 2021).

## 3 Results

### 3.1 County characteristics

**Fig. 2:**
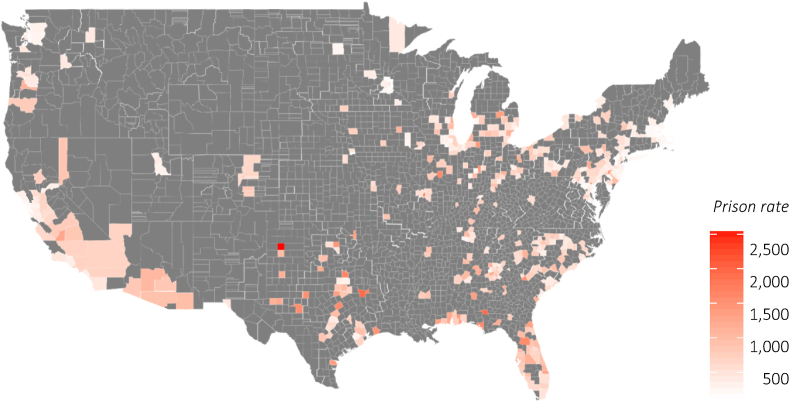
Prison rate heat map for counties included in analyses.

**Fig. 3:**
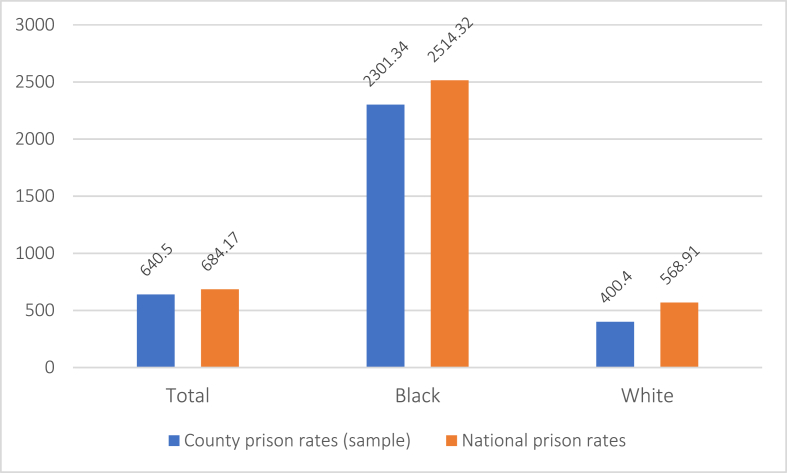
National prison rates vs. average prison rates across sample county populations (N = 475) and stratified by race.

Our data set included a total of 475 counties, excluding 91 with fewer than 50 births in 2016. This represented approximately 67.9% of all live births in the US in 2016 (Hamilton, Martine, Osterman, Driscoll, & Rossen, 2017). Counties included in this study were concentrated mainly in the Eastern and Southern U.S. (see Fig. 2). Total county prison rates averaged 640.50 (median = 596.46) per 100,000 capita, ranging from 91.94 to 2540.62 (Table 1). Black people were heavily overrepresented at a rate of 2301.34 – over 3.5 times higher than the average and nearly six times higher than the prison rate for White people (Fig. 3). Counties with lowest vs. highest prison rates similarly differed on core socio-demographic variables. Counties with the highest prison rates (above the 80th percentile) in 2014 had a higher than (the national) average proportion of Black residents (18.20% vs. 12.40%) residents living in poverty (25.66% vs. 14.80%). By contrast, counties with the lowest prison rates (below the 20th percentile) had larger than average White populations (73.35% vs. 62.10%) and markedly smaller than average Black populations (7.11% vs. 12.40%). The proportion of people in poverty in these counties was 12.93% (nearly 2% lower than the national mean). Across quartiles, these patterns followed a dose-response-style relation between county prison rates and both county population racial makeup and poverty.

**Table 1:** Descriptive statistics for all variables.

	County N	Mean	SD	Min.	Max.
Prison rate (total)	418	640.50	349.96	91.94	2540.62
Prison rate (Black)	418	2301.34	1291.29	330.90	9909.69
Prison rate (White)	418	400.40	255.48	52.74	2115.70
LBW % (total)	363	7.13	2.11	3.10	16.50
LBW % (Black)	265	11.19	2.14	4.70	18.00
LBW % (White)	340	5.14	.97	3.00	9.20
PTB % (total)	337	8.46	1.69	4.90	16.60
PTB % (Black)	216	11.21	2.26	5.80	17.90
PTB % (White)	325	7.01	1.18	4.00	12.40
IM % (total)	170	6.52	1.82	3.07	13.95
Community mental distress*	471	3.72	.43	2.50	4.90
PCP rate	471	77.42	30.20	16.40	228.50
MHP rate	471	181.30	114.40	19.9	779.70
Uninsured %	475	12.53	5.26	2.62	30.55
High School grad %	475	84.21	6.91	59.90	97.00
Poverty %	475	20.41	7.55	4.40	46.60
Social capital index	475	−0.57	0.61	−2.39	3.34
Resid. Segregation B/W	475	49.80	12.18	18.00	81.00
Black population %	475	13.79	12.41	0.60	70.50
White population %	475	66.63	17.40	10.20	94.30

Note. Rates are per 100,000 capita. * Number of mentally unhealthy days in last month.

**Fig. 4:**
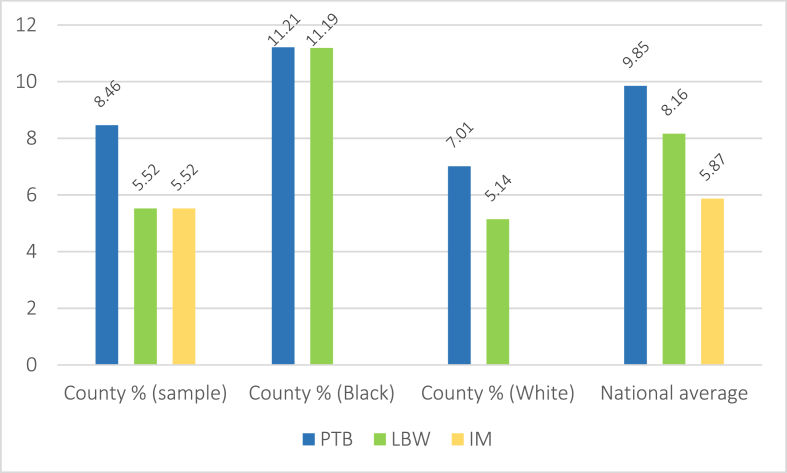
Adverse birth outcome averages across included county populations (N = 475) and stratified by race.

The corresponding county statistics for each of the outcome variables followed a similar pattern. Total county IM and LBW rates averaged 6.52 and 7.13, respectively, with PTB somewhat higher at 8.46. Across all counties, Black people were substantially overrepresented in LBW and PTB, averaging 11.19 and 11.21, respectively, compared to 5.14 and 7.01 for Whites, and 8.16 and 9.85 nationally (Figure 4). Further, higher poverty rates and Black residents were disproportionately concentrated in counties with the highest frequency (80th percentile) of adverse birth outcomes. By contrast, lower poverty rates and White residents were overrepresented in counties with the lowest rates of adverse birth outcomes (20th percentile) (Table 2). We note that because of data suppression regulations, the Ns for each of our outcomes were somewhat lower than the total county sample (LBW = 326, PTB = 297, IM = 170).

**Table 2:** County demographics by quartile of prison rates and adverse birth outcomes.

Prison rate quartile (N)	Variable	Mean	SD	PTB % quartile (N)	Variable	Mean	SD
1 (104)	% NH White	71.94	16.62	1 (84)	% NH White	72.22	17.96
	% NH Black	7.39	6.17		% NH Black	6.29	5.47
	% Poverty	12.93	9.16		% Poverty	16.49	6.91
2 (105)	% NH White	69.28	17.94	2 (84)	% NH White	66.79	18.40
	% NH Black	11.21	9.86		% NH Black	10.20	8.52
	% Poverty	19.82	5.83		% Poverty	20.13	6.94
3 (105)	% NH White	66.03	17.01	3 (85)	% NH White	66.28	16.16
	% NH Black	14.71	11.83		% NH Black	15.26	12.86
	% Poverty	23.06	5.94		% Poverty	21.05	7.05
4 (104)	% NH White	62.42	16.66	4 (84)	% NH White	69.32	14.45
	% NH Black	18.33	14.93		% NH Black	18.55	14.52
	% Poverty	25.66	5.47		% Poverty	22.84	6.90
LBW % quartile (N)				IM % quartile (N)			
1 (91)	% NH White	75.56	16.82	1 (47)	% NH White	56.23	17.45
	% NH Black	4.67	3.73		% NH Black	7.98	5.79
	% Poverty	16.54	6.97		% Poverty	16.65	6.61
2 (92)	% NH White	70.83	17.58	2 (47)	% NH White	58.03	16.67
	% NH Black	8.85	8.12		% NH Black	13.03	9.35
	% Poverty	18.48	6.78		% Poverty	19.98	6.19
3 (88)	% NH White	66.28	16.62	3 (48)	% NH White	63.69	16.63
	% NH Black	15.17	11.74		% NH Black	14.74	11.53
	% Poverty	21.24	6.84		% Poverty	22.22	5.99
4 (92)	% NH White	68.26	13.17	4 (47)	% NH White	57.33	17.56
	% NH Black	20.00	13.58		% NH Black	28.36	16.75
	% Poverty	23.80	6.02		% Poverty	26.54	6.69

Note. Quartiles are in ascending order.

### 3.2 Regression analyses

To test H_1_, we regressed county-level IM, LBW, and PTB onto prison rates in separate analyses and adjusted for all covariates. Initial scatter plots, residual plots, and Q-Q plots confirmed assumptions of linearity, homoscedasticity, and normality. Durbin-Watson test results similarly indicated independence of residuals for each of our analyses. All regression results are presented in Table 3.

**Table 3:** Adjusted and unadjusted regression analyses.

Model 1 (unadjusted)						Model 2 (adjusted)				
**Infant mortality (N** = **170)**	β	*t*	*p*	95% LCI	95% UCI	β	*t*	*p*	95% LCI	95% UCI
County prison rate	.60	9.76	<.001	.002	.004	.40	5.17	<.001	.001	.003
Poverty						.12	1.38	.170	−.012	.067
Social capital						.12	1.94	.060	−.006	.610
% county population Black						.34	5.18	<.001	.026	.059
Res. Segregation (Black/White)						−.02	−.02	.737	−.021	.015
High school grad rate						.04	.53	.598	−.022	.038
**LBW (N** = **326)**										
County prison rate	.39	7.61	<.001	.002	.003	.14	2.21	.028	.000	.002
Poverty						.18	2.53	.012	.012	.092
Social capital						.03	.58	.563	−.250	.459
% county population Black						.37	6.91	<.001	.050	.089
Res. Segregation (Black/White)						−.08	−1.65	.100	−.032	.003
High school grad rate						.06	1.05	.294	−.015	.051
**PTB (N** = **297)**										
County prison rate	.36	6.67	<.001	.001	.002	.17	2.40	.017	.000	.001
Poverty						.14	1.82	.069	−.003	.066
Social capital						−.03	−.52	.607	−.379	.222
% county population Black						.34	5.94	<.001	.033	.066
Res. Segregation (Black/White)						−.10	−1.81	.071	−.028	.001
High school grad rate						.08	1.40	.164	−.008	.049

The unadjusted models revealed that greater prison rates were associated with increases in each of the three outcomes. All effect sizes were medium (LBW β = 0.39, 95% CI = 0.002, 0.003; PTB β = .36, 95% CI = 0.001, 0.002) to strong (IM β = 0.60, 95% CI = 0.002, 0.004). After adjusting for all covariates, these effect sizes remained statistically significant.

### 3.3 Mediation analyses

**Fig. 5:**
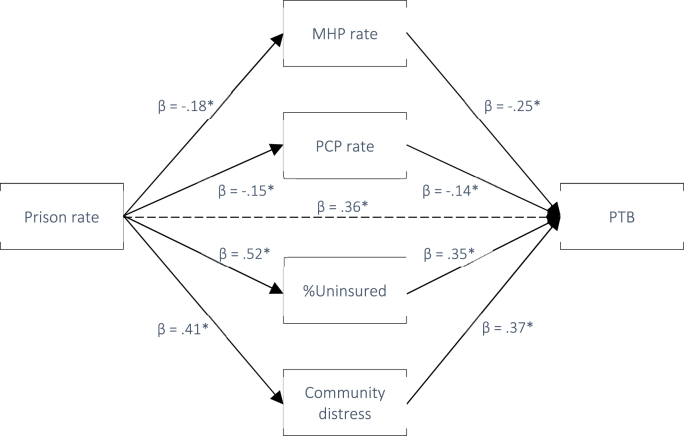
Pathway diagram for the association between Prison rate and PTB with standardized beta weights (* = p < .001).

**Fig. 6:**
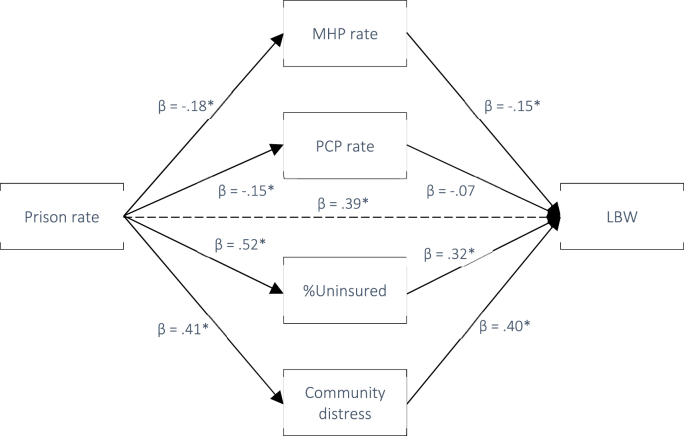
Pathway diagram for the association between Prison rate and LBW with standardized beta weights (* = p < .001).

**Fig. 7:**
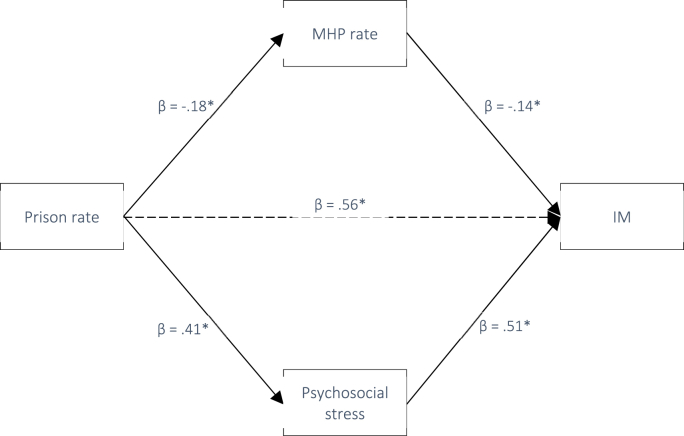
Pathway diagram for the association between prison rate and IM with standardized beta weights (* = p < .001).

In line with H_2_ and H_3_, we next tested whether the associations between county-level prison rates and LBW, PTB, and IM were mediated by community-level mental distress and/or county healthcare access. Pathway statistics for the exposures and outcomes are provided in Table 4. All pathways were significant except for *b*_*1*_ between PCP rate and LBW and IM. According to current recommendations, this does not preclude potential mediation as the indirect effect represents the *product* of pathway *a*_*1*_ and *b*_*1*_ on the outcome and therefore does not require statistically significant individual *a*_*1*_ and *b*_*1*_ paths (Hayes, 2022). Standardized indirect effects at each level of exposure and outcome (i.e., the overall association between prison rate and each birth outcome) are presented in Table 5 and depicted in Fig. 5, Fig. 6, Fig. 7.

**Table 4:** Pathways from exposure to mediator, and from mediator to outcomes. *Note.* Ns for each mediation analysis are defined by outcome (LBW = 326, PTB = 297, IM = 6.52).

Path *a*_*1*_: Exposure to mediators						
Exposure	Mediator	β	*t*	*p*	95% LCI	95% UCI
Prison rate	MHP	−.18	−3.70	<.001	−.090	−.028
	PCP	−.15	−3.15	.002	−.021	−.005
	%Uninsured	.51	12.08	<.001	.007	.009
	Mental distress	.41	9.08	<.001	.000	.001

Path *b*_*1*_: Mediators to outcomes						
Mediators	Outcome					
MHP	PTB	−.25	−4.60	<.001	−.005	−.002
	LBW	−.15	−2.97	.003	−.005	−.001
	IM	−.14	−3.00	.003	−.004	−.001
PCP	PTB	−.14	−2.66	.008	−.014	−.002
	LBW	−.07	−1.25	.213	−.012	.003
	IM	−.11	−1.52	.129	−.009	.002
%Uninsured	PTB	.35	6.87	<.001	.092	.166
	LBW	.32	6.50	<.001	.140	.195
	IM	.29	6.47	<.001	.069	.129
Community distress	PTB	.37	7.30	<.001	1.068	1.857
	LBW	.40	8.27	<.001	1.510	2.452
	IM	.51	12.67	<.001	1.812	2.478

**Table 5:** Indirect effects of exposure on outcome via each mediator.

Exposure	Outcome (N)	Mediator	Std. Effect	Boot SE	95% LCI	95% UCI
Prison rate	PTB (297)	MHP rate	.0385^a^^	.0156	.0116	.0723
		PCP rate	.0195^a^^	.0119	.0011	.0489
		Uninsured	.1024^a^^	.0312	.0490	.1735
		Community distress	.1121^a^^	.0420	.0358	.1998
Prison rate	LBW (326)	MHP rate	.0141	.0102	−.0012	.0390
		PCP rate	.0016	.0110	−.0201	.0250
		Uninsured	.0697^a^^	.0278	.0196	.1281
		Community distress	.1278^a^^	.0321	.0692	.1959
Prison rate	IM (170)	MHP rate	.0264^a^^	.0179	.0015	.0719
		PCP rate	.0075	.0132	−.0128	.0420
		Uninsured	−.0170	.0334	−.0828	.0502
		Community distress	.1302^a^	.0373	.0624	.2086

a = statistical significance; ^ = statistical significance in adjusted model.

Our unadjusted results indicated that the association between greater county prison rates and increased LBW rates was mediated by community-level mental distress (indirect effect (IE) = 0.1278, 95%CI 0.0692, 0.1959) and %Uninsured (IE = 0.0697, 95%CI 0.0196, 0.1281). No statistically significant mediation was detected for MHP rate (IE = 0.0141, 95%CI -0.0012, 0.0390) or PCP rate (IE = 0.0016, 95%CI -0.0201, 0.0250). Adjusting for covariates, the mediation effects for %Uninsured and community-level mental distress were sustained.

Next, unadjusted mediation models revealed that the positive association between county prison rates and PTB was mediated by MHP rate (IE = 0.0385, 95%CI 0.0116, 0.0723), PCP rate (IE = 0.0195, 95%CI 0.0011, 0.0489), %Uninsured (IE = 0.1024, 95%CI 0.0490, 0.1735), as well as community-level mental distress (IE = 0.1121, 95%CI 0.0358, 0.1998). These effects remained in the adjusted models.

Finally, in terms of IM, the unadjusted models supported mediation of the association between county prison rate and IM via MHP rate (IE = 0.0264, 95%CI 0.0015, 0.0719) and community-level mental distress (IE = 0.1302, 95%CI 0.0624, 0.2086). The effect for MHP (but not community-level mental distress) remained in the adjusted model (Table 5).

## 4 Discussion

Our results confirm the positive association between county-level incarceration rates and adverse birth outcomes. These associations were robust to the inclusion of carefully selected evidence-based covariates – including poverty, education, residential segregation, and social capital – and strongly suggest that spatial concentrations of mass incarceration are uniquely associated with incidence rates of adverse birth outcomes. This corresponds with past studies on this topic discussed in the introduction (Chambers et al., 2018; Chambers et al., 2019; Jahn et al., 2020; Holaday et al., 2021; Montgomery et al.). Importantly, however, while previous research offers important contributions to the literature, none have conducted analyses on potential pathways. Filling this gap, our study offers new evidence that multiple pathways may underpin the association between mass incarceration and adverse birth outcomes. Specifically, our findings suggest that concentrated incarceration may contribute to an increase in community-level mental distress and reduced access to health care insurance, PCPs, and MHPs which in turn may increase the risk of population-level adverse birth outcomes. These associations were consistent across mediators for the link between prison rates and PTB. Health insurance and community-level mental distress – but not PCP or MHP – also mediated the association between prison rates on LBW, while only MHP and community-level mental distress mediated IM. These results may thus contribute to a deeper understanding of how spatial concentrations of incarceration negatively impact an area as well as its community and social infrastructure, with severe repercussions for rates of adverse birth outcomes.

Our findings also point to the intergenerational harm that may be caused by mass incarceration and structural racism. As noted, adverse birth outcomes are associated with increased risk of neurodevelopmental delays and disabilities (Luu, Mian, & Nuyt, 2017; Schieve et al., 2016), poor cardiovascular health (Allen, Oluwafemi, & Patel, 2023; Nordman, Jääskeläinen, & Voutilainen, 2020), and premature mortality (Callaghan et al., 2006). While these intergenerational effects are poorly understood, our work emphasizes community-level mental distress and health care access as important health-risk factors that may mediate both present and future community health. Further, our study also aligns with burgeoning evidence of other community-level health repercussions of concentrated incarceration, including increased mortality rates (Kajeepeta et al., 2021) and prevalence of cardiometabolic disease (Topel et al., 2018), asthma (Frank et al., 2013), sexually transmitted infections (Dauria et al., 2015), and mental health (Hickson et al., 2022). While empirical research into the mechanisms of these particular associations is lacking, our mediation results for birth outcomes may well generalize to these other contexts as well. For example, multiple studies have discussed both stress proliferation and spatial/logistical access barriers to health care as key risk factors for many of these community-level health outcomes (Fahrenbach et al., 2020; Hickson et al., 2022; Janevic et al., 2020; Largent, 2018; Tung et al., 2019). Our study bolsters and advances this line of research with strong empirical evidence.

### 4.1 Practical implications

In light of these findings and the broader evidence base, we suggest key areas for future health policy research. We recognize that a multi-pronged approach is necessary to encourage individual- and structural-level interventions to facilitate healthy pregnancies and reduce Black-White maternal health disparities in the U.S.

First, the practice of mass incarceration needs to end and racial inequities in imprisonment must be eliminated. This requires a concerted and consistent effort on multiple fronts, including dismantling racist upstream policies such as the ‘wars’ on drugs and poverty, as well as purging pervasive discriminatory practices in the criminal justice system (e.g., systems of bail, three-strikes sentencing laws), and replacing punitive policing strategies (e.g., stop-and-frisk zones, reasonable suspicion, etc.) with improved diversion programs and proactive community policing (Clear, 2009; Simes, 2018).

Second, eliminate prison gerrymandering. When individuals are incarcerated they are typically removed from their communities and relocated to state or federal facilities in other districts where they are counted in the U.S. census as residents. The mass removal of people may thus have wide-ranging ramifications for the distribution of infrastructural funding and resources (including health care) in areas affected by concentrated incarceration (Calabrese, Gordon, & Lu, 2023; Clear, 2009). While momentum to abandon prison gerrymandering is growing (Golembeski et al., 2008; Walker et al., 2017), only 17 states have currently passed legislation to this effect (Kajstura, 2023). Compounding the impact of prison gerrymandering, in all but two states people with histories of incarceration are subject to restricted voting rights, further muting community political capital and increasing exposure to poor health. Indeed, acknowledging research on the community health effects of civic engagement, Healthy People 2030 has included voting as a determinant of health and an objective for general expansion by 2030 (Brown, Raza, & Pinto, 2020; Social and Community Context, 2023). Thus, eliminating prison gerrymandering and restoring unrestricted voting rights for people with histories of incarceration could help return political and social capital to communities affected by mass incarceration and improve infrastructural resources and community health.

Third, institute “ban-the-box” legislation. “Ban-the-box” legislation prohibits employers from asking job applicants about incarceration history in their initial application. A criminal record poses a significant barrier to food and housing assistance, education, employment, and voting (Bandara, McGinty, & Barry, 2020; Eberstadt, 2019). A recent report found that people with incarceration and/or criminal conviction histories lost an estimated collective $372,300,000,000 in annual earnings in 2017. These economic losses are nearly 3.3 times higher for Black and Latinx people compared to White people, thus contributing significantly to the racialized wealth gap and, by extension, to community stress and barriers to quality health care (our tested pathways to birth outcomes) (Craigie, Grawert, & Kimble, 2020; Paul et al., 2009). Research suggests that prohibiting employers from requesting history of incarceration from applicants as part of the “ban-the-box” employment policies, could lead to community-level improvements. For example, one study found that for people with conviction histories, this initiative increased the likelihood of obtaining employment by about 30% (Craigie, 2020). In spite of such findings, these policies do not extend across the country, there is generally less public support, and the majority of initiatives are applicable to federal, not private sector employment (Avery, 2019). On balance, studies have also shown that these policies can increase discrimination such that White applicants are more likely to benefit than Black applicants (Doleac, 2019). Thus, while “ban-the-box” represents a potential intervention and policy change that could reduce inequities, further research is needed on exactly how this might best be implemented.

Finally, implement national screening for depression and psychological distress and provide culturally responsive and accessible mental health services (Novacek et al., 2020). The U.S. Preventive Services Task Force and the American Congress of Obstetricians and Gynecologists recommend screening for depression during the perinatal period (Siu et al., 2016). While research suggests that screening is not standardized (Siu et al., 2016), the 2014 Affordable Care Act (ACA) medicaid expansions appear to have improved access to care for reproductive-aged women with low incomes, including increased access to physicians and fewer cost barriers to receiving care (Johnston et al., 2018). These expansions have been associated with a 16% decrease in prepregnacy depression and significant declines in Black infant mortality (Constantin et al., 2023; Margerison et al., 2021). As such, the promotion and expansion of the ACA, may be an important mechanism for supporting screening and other preventive measures to address unmet mental health needs during preconception and antenatal pregnancy periods in communities experiencing mass incarceration.

### 4.2 Strenghts and limitations

There are several methodological and conceptual strengths associated with this study. We curated and linked representative data from multiple reputable sources, including the CDC and the US Census Bureau. As such, the data from which our results are calculated represents some of the highest-quality publicly available data on health outcomes, social determinants of health, and prison rates in the U.S. The fact that we were able to link data across three years and create temporal separation between exposure, mediators, and outcomes, also strengthens our mediation analyses. Further, our pathway variables were based on evidence that strongly suggests (but does not test) their mediating properties.

There are also several limitations that should be noted. First, the lagged cross-sectional design afforded temporal separation between our exposure, mediator, and outcome variables, but falls short of an actual longitudinal analysis. We did not conduct a longitudinal mediation analysis due to lacking data availability pre and post 2015. Specifically, MHP data was not available for 2014. Further, the methodology for collecting MHP and PCP data has not been uniform over time (e.g., definitions of active NPIs and PCPs varies across years), confounding reliable temporal tracking and any longitudinal applications of these measures (AHRF and Area Health Resources Files (AHRF), 2014–2016; CMS, 2022; CHR&R, 2010–2020). Finally, the public access VIJ prison rates data that comprised our exposure variable were only available up to and including 2016, thus restricting data points beyond this vintage. Given this, we cannot draw any firm conclusions about cause and effect. On this point, we also note the lack of controls for stable between-county differences on our outcomes, which could lead to spuriousness in the observed association between prison rates and birth outcomes. Second, due to privacy regulations, much of our data was suppressed with our ultimate sample comprising 475 counties, representing 15% of all US counties (N = 3143). While descriptive statistics indicate that our sample matches up well against the general population, including more counties in our analyses would have improved statistical power and potentially clarified some of our more unstable results (e.g., the mediating effect of PCP density). Third, publicly available birth data is derived from birth certificates or other government documentation which is not of the same quality or level of detail as actual clinical records. For this reason, there is a margin of error that should be taken into account when interpreting this data. Similarly, our data did not include maternal factors other than nulliparity. Because of this, we were unable to control for individual-level maternal health-risk factors or comorbidities. Other data limitations relate to the mediator variables. For example, PCP rate precludes other primary care services such as physician assistants or nurse practitioners and thus may not reflect the full extent of available health care. Further, PCPs and MHPs who operate on the edge of county lines may service people from multiple counties. This data may thus underestimate actual health care accessibility. It should also be noted that health care density provides no information about the quality of care. Finally, the measure of community distress was based on self-report survey data and thus vulnerable to limitations inherent to this methodology (bias, response rate, self-selection). We also note that the mediators tested in this study presumably represent only a few of the total number of pathways that connect mass incarceration and adverse birth outcomes (e.g., police victimization, crime rate, etc.). However, because of lacking county-level data availability on other theorized mediators, we were confined to include only health care access and community distress. Future work with quality datasets will continue to advance our understanding of these complex relationships.

## 5 Conclusion

Despite decades documenting the Black-white disparities in adverse birth outcomes, implementation strategies of evidence-based interventions have not been optimal. In understanding the complexity of reproductive health inequities rooted in structural racism, we found that county-level racialized mass incarceration is associated with county-level adverse birth outcomes. We also found that these associations were mediated by community mental distress and access to health care. To adequately address inequities in adverse birth outcomes, states and local governments should consider interventions focused on decreasing mass incarceration rates and county-level mental distress, and increasing access to healthcare for residents. To this end, we urge more research – particularly at smaller geographic levels – into the specific causal pathways that lead from spatial concentrations of incarceration to adverse births and other health outcomes.

## Funding sources

Mia Charifson is supported by the 10.13039/100000001National Science Foundation Graduate Research Fellowship Program (RN Grant ID: 20-A0-00-1005789). Any opinions, findings, and conclusions or recommendations expressed in this material are those of the authors and do not necessarily reflect the views of the National Science Foundation or the National Institutes of Health.

## Author contributions

ALS: Conceptualization, Data curation, Formal analysis, Methodology, Visualization, Writing – original draft, Writing – review/editing. NJW: Conceptualization, Methodology, Visualization, Writing – original draft, Writing – review/editing. MC: Conceptualization, Formal analysis, Methodology, Visualization, Writing – review/editing. RO: Methodology, Visualization, Writing – review/editing. SSJ: Methodology, Visualization, Writing – review/editing. EDL: Methodology, Writing – review/editing. AS: Conceptualization, Methodology, Visualization, Writing – review/editing.

## Declaration of competing interest

The authors declare that they have no known competing financial interests or personal relationships that could have appeared to influence the work reported in this paper.

## Data Availability

All data used for this paper is publicly available and accessible online.
